# Increased Mitochondrial Mass and Cytosolic Redox Imbalance in Hippocampal Astrocytes of a Mouse Model of Rett Syndrome: Subcellular Changes Revealed by Ratiometric Imaging of JC-1 and roGFP1 Fluorescence

**DOI:** 10.1155/2017/3064016

**Published:** 2017-08-13

**Authors:** Dörthe F. Bebensee, Karolina Can, Michael Müller

**Affiliations:** ^1^Center for Nanoscale Microscopy and Molecular Physiology of the Brain (CNMPB), Georg-August-Universität Göttingen, Universitätsmedizin Göttingen, Göttingen, Germany; ^2^Zentrum Physiologie und Pathophysiologie, Institut für Neuro- und Sinnesphysiologie, Humboldtallee, 23 Göttingen, Germany

## Abstract

Rett syndrome (RTT) is a neurodevelopmental disorder with mutations in the *MECP2* gene. Mostly girls are affected, and an apparently normal development is followed by cognitive impairment, motor dysfunction, epilepsy, and irregular breathing. Various indications suggest mitochondrial dysfunction. In Rett mice, brain ATP levels are reduced, mitochondria are leaking protons, and respiratory complexes are dysregulated. Furthermore, we found in MeCP2-deficient mouse (*Mecp2^−/y^*) hippocampus an intensified mitochondrial metabolism and ROS generation. We now used emission ratiometric 2-photon imaging to assess mitochondrial morphology, mass, and membrane potential (ΔΨm) in *Mecp2^−/y^* hippocampal astrocytes. Cultured astrocytes were labeled with the ΔΨm marker JC-1, and semiautomated analyses yielded the number of mitochondria per cell, their morphology, and ΔΨm. *Mecp2^−/y^* astrocytes contained more mitochondria than wild-type (WT) cells and were more oxidized. Mitochondrial size, ΔΨm, and vulnerability to pharmacological challenge did not differ. The antioxidant Trolox opposed the oxidative burden and decreased the mitochondrial mass, thereby dampening the differences among WT and *Mecp2^−/y^* astrocytes; mitochondrial size and ΔΨm were not markedly affected. In conclusion, mitochondrial alterations and redox imbalance in RTT also involve astrocytes. Mitochondria are more numerous in *Mecp2^−/y^* than in WT astrocytes. As this genotypic difference is abolished by Trolox, it seems linked to the oxidative stress in RTT.

## 1. Introduction

Rett syndrome (RTT) is a postnatal, X-chromosome linked, progressive neurodevelopmental disorder. The first RTT cases were reported in 1966 by the Austrian pediatrician Andreas Rett [1]; the characteristic symptoms include autistic features, dementia, motor dysfunction, loss of facial expressions, stereotypical hand movements, severe respiratory disturbances, and epilepsy [1–3]. RTT almost exclusively affects girls, typically at an incidence of 1 : 10,000–15,000 [4]. In boys, it is either lethal or due to severe neonatal encephalopathy death occurs during the first year [5]. Despite being a rare disease, RTT—next to Down syndrome—is the second most common genetic cause of severe cognitive disabilities in girls [4].

The main causes of classical RTT are de novo mutations of the *MECP2* (methyl-CpG-binding protein 2) gene. It is located on the long arm of the X chromosome (Xq28) [6] and functions as a transcriptional modulator by either mediating gene silencing [7] or acting as transcriptional activator [8]. The spontaneous *MECP2* mutations underlying RTT mostly occur in the paternal X chromosome [9, 10]. In the affected girls, the disease manifests in early childhood and progresses in four stages: An initial and apparently normal development (stage 1) is followed by a fast regression (stage 2), and then, a plateau or pseudostationary phase manifests (phase 3), which is concluded by a late motor deterioration (phase 4) [3, 11]. The life expectancy varies with clinical severity, and some Rett patients may reach the age of 70 years [12]. Often, death arises from cardiac/respiratory insufficiency, acute infections, or sudden incidents at night [12, 13]. A causal therapy for RTT is currently not available. Yet, some symptoms can be partly ameliorated by pharmacotherapy [14, 15], occupational therapy, and/or physical therapy [16, 17].

The past years revealed that RTT is closely associated with mitochondrial alterations, and in view of the multitude of mitochondrial functions, these defects were proposed to contribute to disease progression [18, 19]. It was even speculated whether RTT may represent a mitochondrial disease [20]. Morphological alterations of mitochondria were first detected in muscle biopsy samples of Rett patients, whose mitochondria appeared swollen or dumbbell-shaped [21] and exhibited granular inclusions, vacuolizations, and membranous changes [22, 23]. Post mortem studies on frontal lobe confirmed structural changes of mitochondria also for the brain [22].

Detailed follow-up biochemical analyses revealed a reduced expression of a subunit of cytochrome c oxidase (complex IV of the respiratory chain) in post mortem frontal cortex [24], which could impair ATP synthesis. Furthermore, comparative gene-array analyses on patient lymphomonoytes indicated a differential expression of various genes pivotal to mitochondrial function and/or organization [25]. In mouse models of RTT, symptomatic animals also showed reduced enzyme activities of respiratory chain complexes II, III, IV, and ANT1, as well as reduced glutathione levels in the brain and/or skeletal muscle [26–29]. Furthermore, the *ANT1* gene encoding the mitochondrial adenine nucleotide translocase is highly upregulated in the *Mecp2^−/y^* mouse brain and Rett patient fibroblasts [30], the latter of which also show clear signs of metabolic mitochondrial dysregulation, oxidative stress, and diminished redox-balancing capabilities [31]. Also, the elevated blood lactate and pyruvate levels suggest defects in the mitochondrial respiratory chain and the urea cycle [32]. Obvious consequence of these mitochondrial changes is a less efficient respiratory chain and thus limited ATP synthesis. Indeed, magnetic resonance tomography and biochemical assays confirmed reduced brain ATP levels in male and female *Mecp2*-mutant mice [26, 33]. In view of the severe respiratory disturbances and frequent apneas in Rett patients [34–36], systemic hypoxia may occur and challenge mitochondrial function further. This led to the assumption that the various mitochondrial abnormalities are causal events in RTT. They may contribute to the complex symptoms of RTT and promote disease progression either directly or indirectly by generating free radicals and provoking redox imbalance [18, 19, 28, 29, 37–40].

The mostly neuronal alterations in RTT [41–43] originally suggested a purely neuronal disorder. Only later glial cells were confirmed to contain MeCP2 and to be affected by *Mecp2* mutations as well [44]. Meanwhile, it is clear that also glial cells contribute to disease progression by exerting negative effects on neurons [45, 46]. In MeCP2-deficient mice, re-expression of MeCP2 specifically in astrocytes improved motor function, respiratory regularity, and anxiety, and the life-expectancy increased significantly as compared to mice lacking MeCP2 [45]. Restoring MeCP2 in oligodendrocytes was also beneficial, ameliorating motor symptoms and normalizing body weights [46]. Also, MeCP2-deficient microglia contribute to disease progression, as its intensified glutamate release provokes dendritic malformations, microtubule derangement, and damage of postsynaptic glutamatergic components [47]. Therefore, we here assessed to what degree also astrocytes show mitochondrial alterations. Using advanced optical tools, we took a closer look at the function and morphology of these organelles in cultured astrocytes of neonatal *Mecp2^−/y^* and WT mice and also quantified cellular redox conditions. Our particular focus was on the hippocampus, as this brain area is metabolically demanding and vulnerable to oxidative stress.

## 2. Materials and Methods

This study was performed on *Mecp2* knockout mice [B6.129P2(C)-Mecp2^tm.1.1Bird^, [48]]. To ensure uniform conditions, that is, total MeCP2 deficiency, only male wild-type (WT) and male Rett mice (*Mecp2^−/y^*) were used. *Mecp2^−/y^* mice show a more severe disease progression and develop earlier symptoms as compared to heterozygous female Rett mice [48]. All procedures met the German regulations and were authorized by the Office of Animal Welfare of the University Medical Center Göttingen.

### 2.1. Solutions

If not mentioned differently, all compounds were purchased from Sigma-Aldrich. The artificial cerebrospinal fluid (ACSF) contained 130 mM NaCl, 3.5 mM KCl, 1.25 mM NaH_2_PO_4_, 24 mM NaHCO_3_, 1.2 mM CaCl_2_, 1.2 mM MgSO_4_, and 10 mM dextrose. It was aerated continuously with carbogen gas mixture (95% O_2_, 5% CO_2_) to maintain a stable pH of 7.4 and a proper O_2_ supply of the cells during the imaging experiments.

Minimum essential cell culture medium (MEM, Invitrogen) was supplemented with 0.2 mg/ml NaHCO_3_, 0.1 mg/ml transferrin (Calbiochem/Merck), and 5 mg/ml glucose. For the initial plating of the cells, it furthermore contained 10% FCS (fetal calf serum, Biochrom), 25 *μ*g/ml insulin, and 2 mM L-glutamine. After the first day in culture, a slightly different medium was used (growth medium), which contained 5% FCS, 0.5 mM L-glutamine, 20 *μ*l/ml B27 50x supplement (Invitrogen), and 100 *μ*g/ml penicillin-streptomycin (Biochrom).

For most drugs, we first prepared stock solutions. Sodium cyanide (CN^−^) was dissolved as 1 M aqueous stock and stored at −20°C; other experimental solutions containing CN^−^ were prepared from this stock right before use. FCCP (carbonylcyanide-4-(trifluoromethoxy) phenylhydrazone, Tocris Bioscience) was dissolved as 10 mM stock in dimethyl sulfoxide (DMSO) and stored at 4°C. The mitochondrial markers JC-1 (5,5′,6,6′-tetrachloro-1,1′,3,3′-tetraethylbenzimidazolylcarbocyanine iodide, Thermo Fisher Scientific) and MitoTracker Red FM (Thermo Fisher Scientific) were dissolved in DMSO as 2 mg/ml and 1 *μ*M stocks, respectively, and kept frozen. The free-radical scavenger Trolox ((±)-6-hydroxy-2,5,7,8-tetramethylchromane-2-carboxylic acid) was first dissolved in DMSO and then added to cell culture medium for overnight treatment.

### 2.2. Preparation of Dissociated Hippocampal Cell Cultures

Cell cultures of hippocampal neurons and glial cells were prepared from 2- to 4-day-old mice as described earlier [49]. In brief, mice were decapitated, the brain was gently but quickly removed and immersed in ice-cold HBSS (Hank's balanced salt solution) containing 20% FCS. Both hippocampi were then isolated, cut with a scalpel into smaller pieces, and trypsinated (5 mg/ml, 10 min, 37°C). The cells were then dissociated by trituration and centrifuged (1500 rpm, 10 min, 4°C), and the pellet obtained was resuspended. Next, this cell suspension was plated on Matrigel (BD Biosciences)-coated glass cover slips, which were placed in 4-well culture plates (Nunc). Dissociated cell cultures were incubated at 37°C (5% CO_2_ atmosphere). After 24 h, the medium was replaced with growth medium, and after another 3 days, growth factors as well as half of the medium were refreshed again. Experiments were performed between 3 and 10 days in vitro.

### 2.3. Multiphoton Imaging of Mitochondrial JC-1 Fluorescence

To visualize mitochondria and monitor their membrane potentials (ΔΨm), the membrane potential probe JC-1 was chosen. JC-1 accumulates in mitochondria depending on their ΔΨm, and it is ratiometric by emission [50–55]. Cells were bulk loaded with JC-1 (1 *μ*g/ml, 15 min) immediately before the experiments.

**Figure 1:**
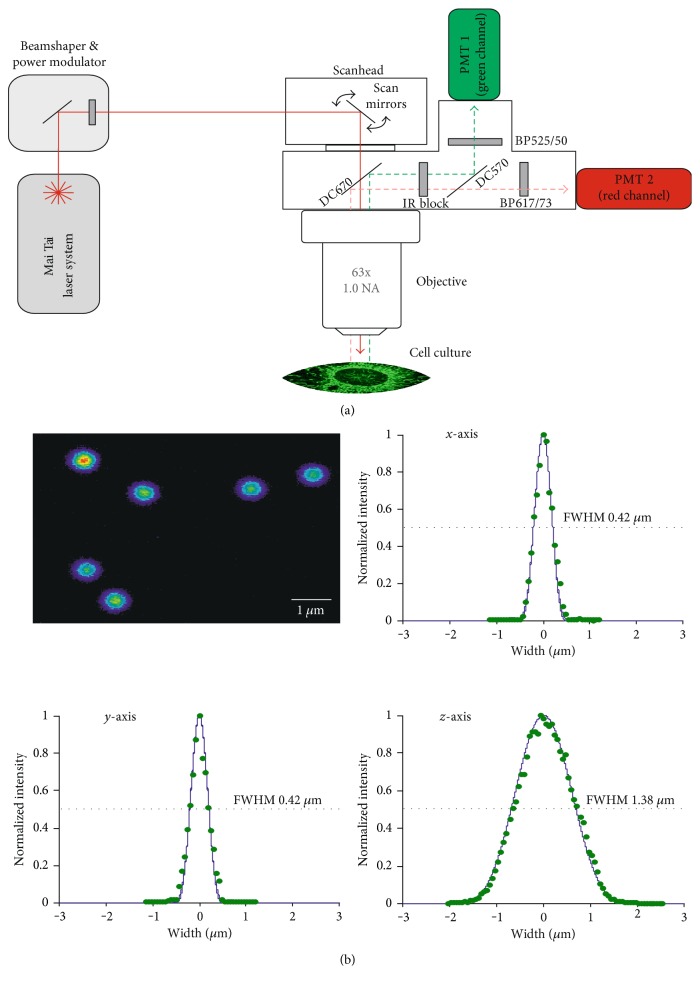
Two-photon laser-scanning microscope (TPLSM) and its point spread functions. (a) General layout of our TPLSM. Fluorescence emission was detected in nondescanned mode by photomultiplier tubes (PMTs). For ratiometric JC-1 imaging, green and red components of JC-1 emission were separated spectrally and detected by the two detection channels. (b) To estimate the spatial resolution, the point spread function of our TPLSM was determined from the intensity profiles of subresolution (100 nm) beads. Displayed intensity profiles are the averages of 24 beads, and excitation wavelength was 800 nm. Their full width at half maximum (FWHM) yields a lateral (X,Y) resolution of 0.4 *μ*m and an axial (Z) resolution of 1.4 *μ*m.

Our 2-photon laser-scanning microscope (TPLSM, Figure 1()) is composed of a Ti : Sa laser system (Mai Tai eHP DS, Newport-Spectra Physics) and an upright microscope (BX51WI, Olympus) equipped with a TriM Scope II scanhead and ImSpector Control Software (LaVision BioTec). To separate the emitted fluorescence and the laser excitation, a 670 nm beam splitter (670DCXXR) was combined with an IR-block filter (HC 680/SP). Fluorescence was detected in nondescanned mode by highly sensitive photomultiplier tubes (PMTs; H7421/H7422, Hamamatsu). For emission ratiometric analyses, JC-1 was excited at 925 nm, and the emission was divided further in its green and red spectral components by using a 570 nm beam splitter (570DCXR) as well as 525/50 nm (green) and 617/73 nm (red) bandpass filters [54]. All optical filters were obtained from AHF Analysentechnik AG (Tübingen, Germany). For optimized optical resolution and detection efficiency, we chose a 63x 1.0 NA water immersion objective (Plan Apochromat VIS-IR, Zeiss).

For the experiments, cell culture cover slips were placed in a submersion-style recording chamber, which was perfused continuously with warm (37°) ACSF at 4 ml/min flow rate. JC-1-labeled cells were allowed to adjust for 15–20 min, and then experiments were started. Imaging of individual cells was performed using a scanfield size of 80 × 80 *μ*m (resolution 80 nm/pixel) and 1200 Hz line-scanning frequency. To reduce pixel noise, 5-fold line averaging was applied. All 3-dimensional (3-d) image stacks were recorded with axial (Z)-increments of 0.25 *μ*m.

Before the analysis, each 3-d stack underwent blind deconvolution (Autodeblur 9.3, AutoQuant Imaging), based on the objective used (1.0 NA) and the center wavelengths of the two different emission channels (525 nm and 617 nm). For all further image analyses, Metamorph Offline 7.5 (Molecular Devices) was used. Background subtraction was not performed during the ratiometric calculations, as in the case of 2-photon excitation, out-of-focus light is virtually nonexisting. However, to ensure a sufficient coverage of the 8-bit pseudocolor palette, a scaling factor of 200 was introduced, and the JC-1 ratio was calculated as

(1) $$\begin{array}{c} R_{JC‐1}=\left(\frac{F_{green\;\left(530\;nm\right)}*200}{F_{red\;\left(590\;nm\right)}}\right). \end{array}$$

### 2.4. Excitation Ratiometric Redox Imaging

Cytosolic redox balance was determined with the excitation ratiometric optical redox indicator roGFP1 (oxidation-reduction sensitive green fluorescent protein 1) [56]. Within 2-3 days upon transfection (lipofection) with roGFP1 expressing plasmids (see [57]), cultured astrocytes sufficiently expressed roGFP1. For excitation-ratiometric analyses, roGFP1 was excited alternately at 395 nm (100 ms exposure) and 470 nm (150 ms exposure) with frame rates of 0.1 Hz. The emission was recorded by a fully computerized CCD-camera imaging system (Polychrome II, TILL Photonics) at 525 nm, using a 63x 1.0 NA objective (Zeiss W-Plan Apochromat VIS-IR) and applying 4 × 4 pixel binning. The roGFP1 fluorescence ratio F_395_/F_470_ was calculated in real time, as described earlier [40, 57–59]. For true quantitation, roGFP1 responses were calibrated to saturating doses of H_2_O_2_ (5 mM, 5 min) and DTT (10 mM, 5 min), to obtain those ratiometric values representing complete sensor oxidation/reduction. Based on these calibrations, the relative degree of roGFP1 oxidation (OxD_roGFP1_) and the reduction potential (E_roGFP1_) was calculated [57, 58, 60, 61] for WT and *Mecp2^−/y^* astrocytes.

### 2.5. MitoTracker-Based Visualization of Mitochondria

Occasionally, we used MitoTracker Red to visualize mitochondria. Bulk-loaded cell cultures (1 *μ*g/mg, 20 min, 37°C) were excited at 550 nm, using the abovementioned CCD-camera imaging system and a 40x 0.8 NA water immersion objective (Zeiss Achroplan). MitoTracker emission was separated by a 570 nm dichroic mirror and a 590 nm longpass emitter, and single images were taken at an exposure time of 300 ms. Pixel binning was not applied.

### 2.6. Statistics

The analyzed neuron/glial cultures were obtained from 14 male WT and 15 male *Mecp2^−/y^* mice. During experiments and data analyses, the genotype was still blinded; genotyping was performed at a later time-point from tail biopsies collected during dissection. Data are shown as mean ± standard deviation, *n* reports the number of astrocytes studied. Depending on the type of experiment, significance of the differences and drug effects observed were determined using either paired or unpaired two-tailed Student's *t*-tests. In the diagrams, genotype-related differences are indicated by asterisks (^∗∗∗^*p* < 0.001, ^∗∗^*p* < 0.01, and ^∗^*p* < 0.05), and genotype-matched differences among recording conditions (drug-induced effects) are marked by crosshatches (^###^*p* < 0.001, ^##^*p* < 0.01, and ^#^*p* < 0.05).

## 3. Results

JC-1 is present as either monomer or J-aggregate. The monomers predominate in depolarized mitochondria and emit green fluorescence (~530 nm). Oligomers (J-aggregates) only form in mitochondria with a ΔΨm < −140 mV and emit red fluorescence (~590 nm) [50–53]. Accordingly, JC-1 is ratiometric by emission, and—as we have shown earlier [54]—the relative green and red components of JC-1 fluorescence allow to distinguish mitochondria with high and low ΔΨm and to detect spontaneous and evoked ΔΨm changes. For the current analyses, an updated and further improved version of our TPLSM [54, 62] was used (Figure 1(a)). Therefore, we first ran initial tests with standardized fluorescent beads, to determine the lateral and axial resolution of the upgraded system (Figure 1(b)).

**Figure 2:**
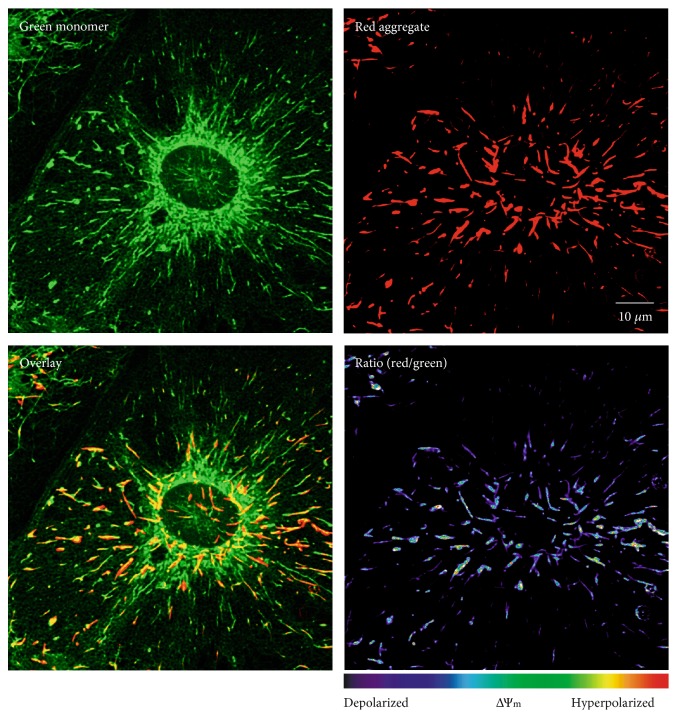
Sample images of a JC-1-labeled hippocampal astrocyte. Displayed are the individual raw images acquired by the two detection channels, representing JC-1 monomers and J-aggregates, respectively. The offline computed overlay image confirms the perfect alignment of the spectrally differing images. The calculated JC-1 ratio (red/green) represents the ΔΨm range of individual mitochondria and their morphological/functional heterogeneity.

To analyze mitochondrial structure and function, cell cultures were bulk loaded with JC-1. Once labeled, astrocytes could be identified clearly by their large cell size and flatly grown shape (Figure 2). Individual astrocytes were then imaged as 3-d image stacks, selecting those cells whose boundaries could be identified clearly and which were not overlapping with other cells. Based on the acquired green and red channels, overlay and ratiometric images were then calculated offline (Figure 2).

To identify potential differences in mitochondrial structure and/or function among WT and *Mecp2^−/y^* astrocytes, these parameters had to be determined for individual mitochondria. Therefore, we developed a semiautomated routine to detect and analyze single particles. First, by blind deconvolution, pixel noise and out-of-focus light were removed, resulting in an improved contrast. Then, the best focal plane of the deconvolved stack was identified by visual inspection and collapsed with its adjacent two upper and two lower planes into a single plane, before thresholding was performed to obtain a binary mask discriminating among mitochondria and background. Spatial filtering then excluded all particles overlapping with others (ramified particles), lying at the margin of the image (truncated particles), or lying outside the size expected for mitochondria (individual bright pixels, or large aggregates in which individual organelles could not be discriminated). The resulting particle mask then included only those mitochondria, which would also be identified visually as individual particles. For each of these particles, the morphological characteristics (particle length) and the functional information (JC-1 ratio) were then extracted from the ratiometric source image.

**Figure 3:**
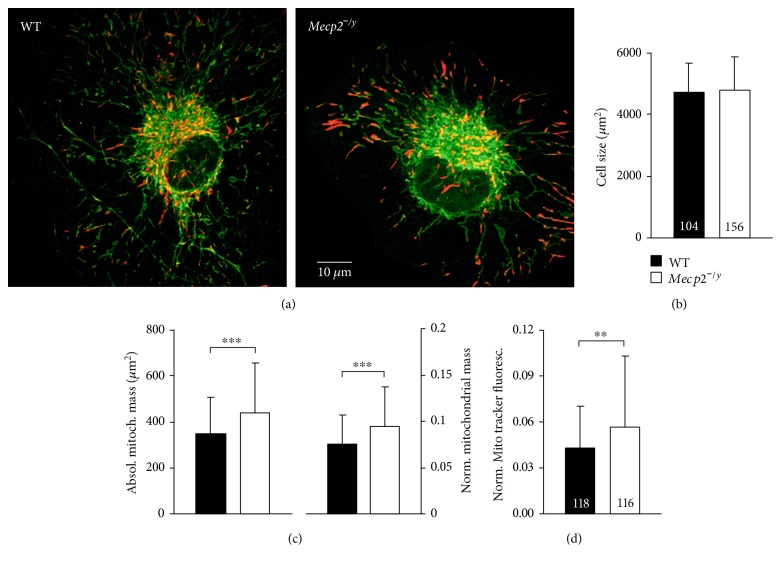
Increased mitochondrial mass in *Mecp2^−/y^* astrocytes. (a) In general appearance, WT and *Mecp2^−/y^* astrocytes did not differ noticeably. Displayed images are overlays of the green and red JC-1 emissions. (b) Astrocytic cell size, determined by circling the outer cell boundaries, was also indistinguishable. Plotted data are mean ± standard deviations, and the number of cells analyzed is included into each bar. Bar shading is identical for the following panels. (c) The absolute mitochondrial mass was higher in *Mecp2^−/y^* astrocytes than in WT cells (left side), and this difference was still present, when the mitochondrial mass was normalized to cell size (right side). Cell numbers analyzed are identical to panel B. Genotypic differences are indicated by asterisks (^∗∗∗^*p* < 0.001). (d) Using the mitochondria-specific marker, MitoTracker Red confirmed the increased mitochondrial mass in *Mecp2^−/y^* astrocytes. Displayed is the normalized intensity of astrocytic MitoTracker Red fluorescence as referred to individual cell size (^∗∗^*p* < 0.01).

In total, 104 WT and 156 *Mecp2^−/y^* astrocytes were imaged and underwent the abovementioned analysis routines. Furthermore, we determined the size of each cell—by circling the outer cell boundaries—as well as the total volume of all JC-1-labeled structures. As total brain size and neuronal complexity differ among WT and Rett mice [42, 63], we first assessed potential genotypic differences in the size of the hippocampal astrocytes. With average cell areas of 4702 ± 950 *μ*m^2^ (*n* = 104) and 4774 ± 1097 *μ*m^2^ (*n* = 156), respectively, cultured WT and *Mecp2^−/y^* astrocytes did not differ at all (Figures 3(a) and 3(b)). Yet, total mitochondrial mass was higher in *Mecp2^−/y^* (440 ± 214 *μ*m^2^) than in WT astrocytes (348 ± 160 *μ*m^2^) (Figure 3(c)). This still was the case, when mitochondrial content was normalized to cell size, yielding an average mitochondrial density of 0.094 ± 0.044/*μ*m^2^ for *Mecp2^−/y^* astrocytes (*n* = 156) and 0.076 ± 0.032/*μ*m^2^ for WT astrocytes (*n* = 104, Figure 3(c)).

To validate this finding, we ran control experiments with the established mitochondrial marker MitoTracker Red [53, 64]. Images of individual astrocytes were acquired with a CCD-camera system, and the cellular intensity of MitoTracker Red fluorescence was normalized to the size of the respective cell. Again, the more intense fluorescence detected in *Mecp2^−/y^* (0.057 ± 0.046; *n* = 116) than in WT astrocytes (0.043 ± 0.028; *n* = 118) indicated a higher mitochondrial content for *Mecp2^−/y^* astrocytes (Figure 3(d)).

**Figure 4:**
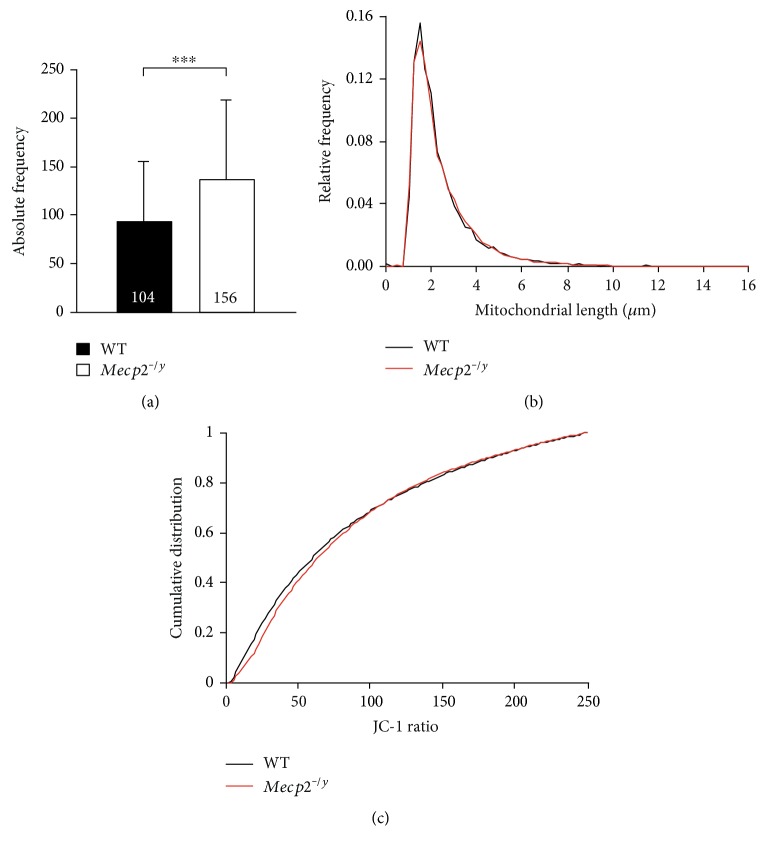
Functional parameters of individual mitochondria. (a) In line with the increased total mitochondrial mass in *Mecp2^−/y^* astrocytes, also more individual organelles could be detected in these cells as compared to WT (^∗∗∗^*p* < 0.001). (b) Genotypic differences in the length of the individual mitochondria were not found. The histogram-type distribution represents the entity of all individual mitochondrial particles detected by the automated analyses in WT (9769 mitochondria) and *Mecp2^−/y^* astrocytes (21,424 mitochondria). (c) The degree of polarization did not differ either among the genotypes. Plotted is the cumulative distribution function of the ΔΨm of individual mitochondria of WT and *Mecp2^−/y^* astrocytes. It is based on the distribution of all recorded JC-1 ratios and indicates on the ordinate the probability that a WT or *Mecp2^−/y^* mitochondrion has a given ΔΨm (or less).

To take a closer look at the genotypic difference in mitochondrial mass, we performed single particle analyses on JC-1-labeled mitochondria. A total of 9769 mitochondria in WT and 21,424 mitochondria in *Mecp2^−/y^* astrocytes were classified, and more individual mitochondria could be detected in *Mecp2^−/y^* (136.5 ± 81.5/cell, *n* = 156) than in WT astrocytes (93.1 ± 6.0/cell, *n* = 104) (Figure 4(a)). Comparing the morphological characteristics, that is, the length of individual mitochondria, did however not reveal any genotypic differences. On average, mitochondria measured 2.34 *μ*m in WT and 3.32 *μ*m in *Mecp2^−/y^* astrocytes (Figure 4(b)). Analyzing the average degree of polarization, assessed as JC-1 ratio, did not reveal any differences either, as indicated by the cumulative distribution function of their ΔΨm (Figure 4(c)). In addition, correlation analysis confirmed that the size of a given mitochondrion does not correlate with its ΔΨm (WT correlation coefficient 0.0895; *Mecp2^−/y^* correlation coefficient 0.103).

### 3.1. Modulation of Mitochondrial Parameters by Radical Scavenger Treatment

Lowered vitamin E levels were found in the blood plasma of Rett patients [65]. Also, we previously confirmed that the vitamin E derivative Trolox improves cellular redox balance as well as synaptic function of the *Mecp2^−/y^* hippocampus in vitro [40, 66], and it ameliorates some RTT symptoms when applied systemically to *Mecp2^−/y^* mice [67]. Therefore, we assessed potential Trolox-mediated effects on mitochondrial function by incubating cell cultures overnight with this free-radical scavenger (100 *μ*M, 12–14 h).

**Figure 5:**
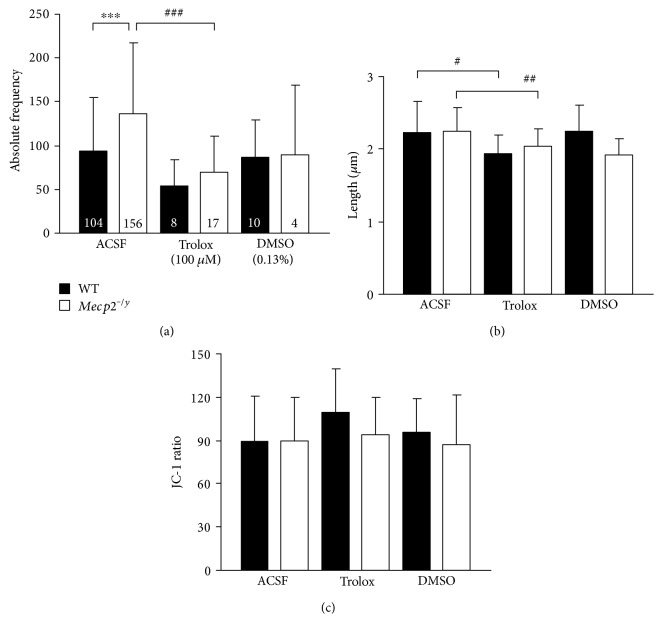
Free-radical scavenger treatment abolishes the differences in mitochondrial mass between WT and *Mecp2^−/y^* astrocytes. (a) Overnight Trolox treatment decreased (or tended to decrease) mitochondrial mass. As a result, the genotypic difference seen under control conditions among WT and *Mecp2^−/y^* astrocytes was no longer detectable. The solvent DMSO itself also tended to decrease the mitochondrial content of *Mecp2^−/y^* astrocytes, but not to the degree seen with Trolox. Bar shading and the number of cells analyzed apply also to the following two panels. Genotpye-related differences are indicated by asterisks (^∗∗∗^*p* < 0.001) and drug-induced genotype-matched differences by crosshatches (^###^*p* < 0.001). (b) Trolox slightly but significantly decreased the length of individual mitochondria to an equal degree in WT and *Mecp2^−/y^* astrocytes. (^#^*p* < 0.05, ^##^*p* < 0.01). (c) Significant changes in ΔΨm could not be observed in response to Trolox treatment. In WT astrocytes, a trend towards increased ΔΨm became obvious though.

Interestingly, Trolox significantly decreased the number of mitochondrial particles in *Mecp2^−/y^* astrocytes and showed a corresponding trend (*p* = 0.076) in WT astrocytes (Figure 5(a)). As a result, the genotypic differences in mitochondrial density among WT and *Mecp2^−/y^* astrocytes were abolished (Figure 5(a)). Part of this effect can also be achieved by the solvent DMSO itself, which also mediates antioxidant capacity by scavenging hydroxyl radicals [68]. Furthermore, in both genotypes, the size of individual mitochondria was slightly smaller in Trolox-incubated than in untreated control cells (Figure 5(b)). Significant effects of Trolox on the ΔΨm of WT or *Mecp2^−/y^* astrocytes were not found, but in WT astrocytes, a trend (*p* = 0.093) towards increased (more negative) ΔΨm became apparent (Figure 5(c)).

### 3.2. Cytosolic Redox Balance

**Figure 6:**
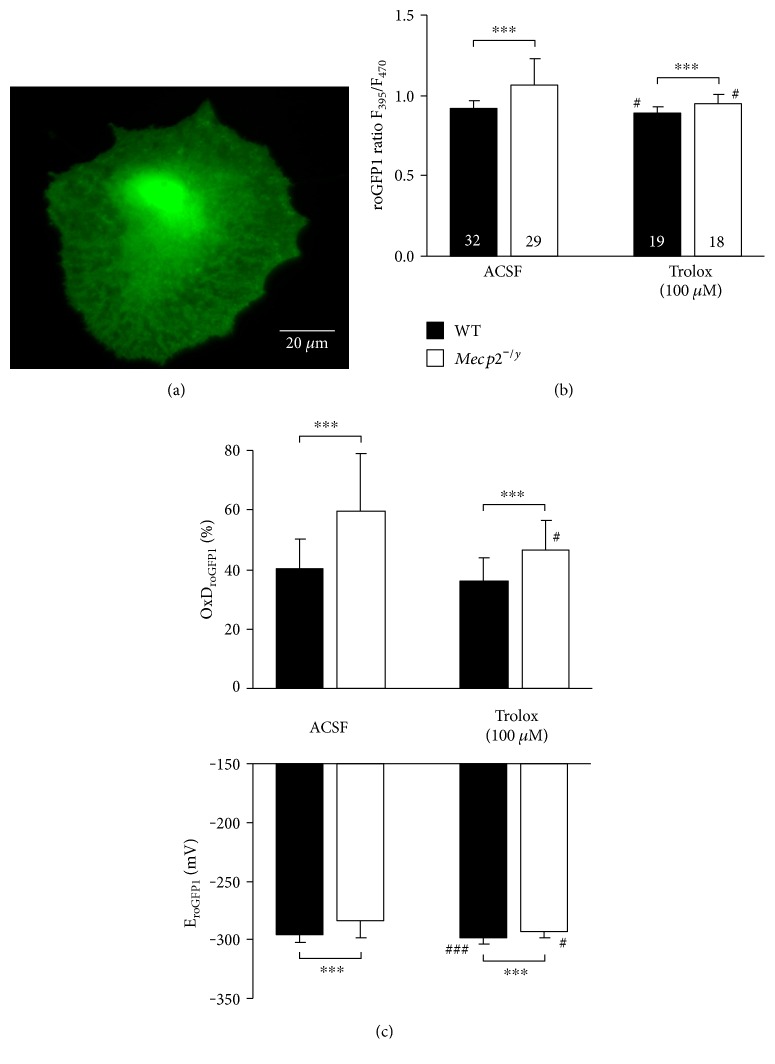
Optical redox imaging with the genetically encoded roGFP1 sensor confirms more oxidized conditions in *Mecp2^−/y^* astrocytes. (a) CCD camera image of a *Mecp2^−/y^* astrocyte expressing cytosolic roGFP1. (b) Under control conditions, fluorescence ratios were higher (more oxidized) in the cytosol of *Mecp2^−/y^* than of WT astrocytes. Trolox induced a shift towards more reducing conditions especially in *Mecp2^−/y^* astrocytes (^∗∗∗^*p* < 0.001, ^#^*p* < 0.05). (c) Calculating the relative degree of roGFP1 oxidation (OxD_roGFP1_) and the corresponding reduction potentials (E_roGFP1_) confirms the more oxidized conditions in *Mecp2^−/y^* astrocytes as well as the antioxidant effect mediated by Trolox (^###^*p* < 0.001).

To confirm the assumption that the differences in mitochondrial mass are closely associated with differences in redox balance among WT and *Mecp2^−/y^* astrocytes, we quantified cellular redox conditions by using the genetically encoded redox sensor roGFP1 (Figure 6(a)). Expressing roGFP1 in the cytosol reported for steady-state resting conditions a fluorescence ratio of 0.92 ± 0.05 (*n* = 32) in WT astrocytes, whereas the fluorescence ratio in *Mecp2^−/y^* astrocytes was slightly higher, that is, more oxidized (1.06 ± 0.17, *n* = 29; Figure 6(b)). This also is evident from the relative degrees of roGFP1 oxidation (WT 40.4 ± 9.8%; *Mecp2^−/y^* 59.7 ± 19.5%) as well as the corresponding reduction potentials (Figure 6(c)). To verify that Trolox (100 *μ*M) improved cellular redox balance, we also quantified cytosolic redox conditions after overnight treatment with this free-radical scavenger. Indeed in WT cells, the relative degree of roGFP1 oxidation slightly decreased to 36.0 ± 7.7% (*n* = 19), whereas it decreased more intensely in *Mecp2^−/y^* astrocytes to 46.7 ± 9.9% (*n* = 18). As a result of this treatment, the genotypic differences in roGFP1 ratio, relative roGFP1 oxidation level, and reduction potential became less pronounced as compared to the untreated astrocytes (Figures 6(b) and 6(c)).

### 3.3. Effects of Mitochondrial Stressors

**Figure 7:**
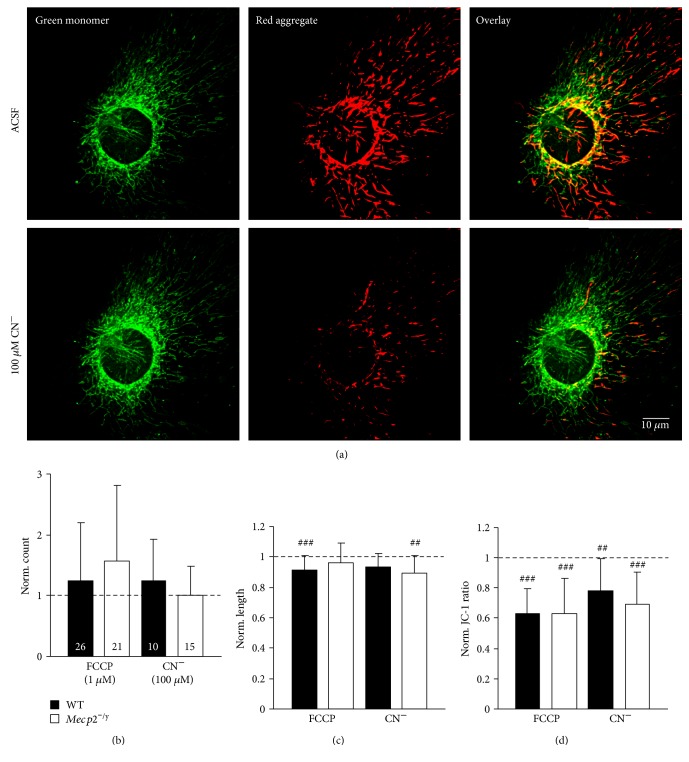
WT and *Mecp2^−/y^* astrocytes show similar vulnerabilities to mitochondrial compromise. (a) CN^−^ treatment (100 *μ*m, 5 min) evoked a clear decrease in JC-1 ratio, which is evident as the green shift of the dual color emission and indicates a marked mitochondrial depolarization; displayed is a WT astrocyte. (b) Significant changes in mitochondrial content (mitochondria/cell) were not evoked by FCCP or CN^−^ treatment. (c) The length of individual mitochondria was affected only very moderately (^##^*p* < 0.01, ^###^*p* < 0.001). (d) As expected, both treatments evoked a clear mitochondrial depolarization. Significant genotypic differences were not observed in response to chemically induced hypoxia (CN^−^) or mitochondrial uncoupling (FCCP).

Previously, we found that the hippocampus of adult *Mecp2^−/y^* mice shows an exaggerated hypoxia susceptibility [69] and also in neonatal hippocampal organotypic slice cultures, we detected exaggerated redox responses to O_2_ shortage [40]. Therefore, we also assessed the effects of mitochondrial stress, by exposing cultured astrocytes to the mitochondrial uncoupler FCCP or by blocking mitochondrial respiration by CN^−^ (Figure 7). To assess the effects of these compounds, each astrocyte was recorded before and after drug treatment, and all drug-induced changes were referred (normalized) to pretreatment control conditions.

Treatment with either FCCP (1 *μ*M, 5 min) or CN^−^ (100 *μ*M, 5 min) markedly decreased the JC-1 fluorescence ratio, indicating a pronounced mitochondrial depolarization (Figures 7(a) and 7(d)). A typical response of mitochondria exposed to such stress is the so-called thread-grain transition, that is, a disruption of longer mitochondrial filaments and a rounding of the individual particles [70–72]. Also here, this was elicited by FCCP in some astrocytes, and it resulted in a markedly increased variability of mitochondrial counts (Figure 7(b)) as well as in a moderately reduced length of the individual mitochondria (Figure 7(c)). As expected, the extent of the FCCP-induced mitochondrial depolarization was identical in WT and *Mecp2^−/y^* cells (Figure 7(d)); this compound acts as protonophore and completely collapses ΔΨm. Also CN^−^ treatment elicited a pronounced mitochondrial depolarization in both genotypes, which tended to be slightly more intense in *Mecp2^−/y^* than in WT astrocytes (Figure 7(d)). Nevertheless, obvious genotypic differences in mitochondrial vulnerability to these pharmacological insults were not detected.

## 4. Discussion

Due to the various alterations of mitochondrial structure and function, which are associated with RTT, mitochondrial dysfunction has been proposed to contribute to disease progression (see [18–20, 28, 29]). Earlier, we found in the hippocampal slices of neonatal *Mecp2^−/y^* mice an increased ratio of FAD/NADH autofluorescence, which indicates an intensified mitochondrial metabolism [40]. This difference was present in *st. pyramidale*, which is largely dominated by pyramidal cell somata, but also in *st. radiatum*, a mixed layer containing neurons and glia. Hence, glial cells may show mitochondrial alterations as well. Therefore, we now conducted optical analyses in WT and *Mecp2^−/y^* glia to characterize further the spectrum of mitochondrial alterations in RTT. Due to their pivotal roles in extracellular ion homeostasis [73], transmitter uptake [74], and blood-brain barrier formation [75], we focused on astrocytes.

The earliest analyses of mitochondria in RTT took advantage of electron microscopy to assess changes in the mitochondrial ultrastructure [20, 22, 76], but this approach lacks functional information. Later, biochemical and molecular biological assays were performed to identify altered enzyme activities and protein levels [23, 25, 29, 77]. Such assays are usually based on full brain or large tissue samples and yield valuable functional insights, but they do not provide single cell resolution. Our 2-photon microscopy study ensured both, functional information as well as subcellular resolution.

Using this technological advantage in combination with the developed semiautomated analysis-routines, we confirmed that mitochondria are more numerous in *Mecp2^−/y^* hippocampal astrocytes. Since the individual mitochondria did not differ in their size and since genotypic differences in cell dimensions were not found, this points out to an increased total mitochondrial mass in *Mecp2^−/y^* astrocytes. One may argue now that the higher number of individual mitochondria identified in cultured *Mecp2^−/y^* astrocytes could arise from a less dense mitochondrial packaging, which facilitated a successful single-particle detection in these cells. Yet, the total mitochondrial content determined in parallel and labeling with the mitochondria-specific marker MitoTracker Red also confirm an increased mitochondrial mass in *Mecp2^−/y^* astrocytes. It therefore seems that the increased mitochondrial content constitutes a cell-endogenous response to compensate for the mitochondria-related deficits and their limited metabolic/respiratory capacity in RTT [26, 27, 31, 40]. Interestingly, an increased mitochondrial mass was also reported for *Mecp2^−/y^* microglia of the juvenile mouse brain [78]. In contrast, *Mecp2*-null mouse skin fibroblasts (cultured in regular medium) and stem-cell derived *Mecp2*-mutant neurons do not differ in their mitochondrial contents [79, 80].

Obvious differences in the size or ΔΨm of individual mitochondria could not be detected among WT and *Mecp2^−/y^* astrocytes. This suggests that the immediate functional impact of the mitochondrial alterations in RTT is rather subtle, which is also indicated by the fact that—in contrast to other mitochondriopathies—a marked degree of neurodegeneration is not evident in RTT [41].

Earlier studies have convincingly linked RTT to cellular redox changes and oxidative stress [37, 40, 81]. The conducted redox analyses now confirm for the first time that the oxidative burden in RTT also applies to astrocytes. As we verified earlier, the free-radical scavenger Trolox improved the cellular redox balance in organotypic hippocampal slices [40], dampened neuronal hyperexcitability in adult hippocampal slices of symptomatic *Mecp2^−/y^* mice, and also improved the hypoxia tolerance as well as certain types of synaptic plasticity [66, 67]. In these studies, any adverse impact of Trolox on mitochondrial function could be ruled out, and also here, an overnight incubation of astrocytes with Trolox did not affect mitochondrial shape or ΔΨm. However, Trolox incubation clearly improved cytosolic redox balance in WT and even more so in *Mecp2^−/y^* astrocytes, thereby dampening the genotypic differences and opposing the oxidative stress in *Mecp2^−/y^* cells. As Trolox also slightly reduced the number of mitochondria per cell and thereby eliminated the genotypic differences seen in untreated astrocytes, it can therefore be concluded that the redox imbalance in RTT is one of the factors that underlies the increased mitochondrial mass in *Mecp2^−/y^* astrocytes.

Furthermore, we assessed potentially different responses of WT and *Mecp2^−/y^* astrocytes to different mitochondria-directed drugs. FCCP evoked marked mitochondrial depolarizations in both genotypes by abolishing the proton gradient across the inner mitochondrial membrane. In addition, it slightly reduced the size of mitochondria in WT astrocytes. As structural changes, or even mitochondrial fragmentation, are quite commonly induced by FCCP [70, 82]; this explains the decreased mitochondrial length and increased number of individual mitochondria detected in some of the WT and *Mecp2^−/y^* astrocytes upon mitochondrial uncoupling. The inhibition of respiratory complex IV by CN^−^ also markedly depolarized mitochondria; the trend towards a more intense depolarization of *Mecp2^−/y^* astrocytes may reflect the increased hypoxia susceptibility we found earlier in the hippocampal and brainstem slices of *Mecp2^−/y^* mice [69, 83–85]. Furthermore, mitochondria of *Mecp2^−/y^* astrocytes became slightly smaller upon CN^−^ treatment. Changes in mitochondrial content were, however, not detectable in any genotype. Therefore, as pronounced genotype-related changes were not detected for mitochondrial size, mitochondrial content, and ΔΨm, it has to be assumed that mitochondria in WT and *Mecp2^−/y^* astrocytes do not noticeably differ in their vulnerabilities to uncoupling and chemical hypoxia. In view of the earlier detected increased hypoxia susceptibility of hippocampal and brainstem networks of MeCP2-deficient mice [69, 85], this is an important finding. Yet, it also has to be considered that the pharmacological challenges applied in the present study were quite intense.

Our 2-photon analyses were based on the emission ratiometric ΔΨm indicator JC-1. We developed and thoroughly tested this JC-1-based emission ratiometric 2-photon imaging approach earlier [54], and it allows not only to visualize individual mitochondria but also to compare their ΔΨm and quantify any ΔΨm changes. JC-1 clearly differs from other dyes, as it is the only emission ratiometric indicator detecting ΔΨm changes with sufficient sensitivity. Often criticized is that JC-1 reacts more slowly than other mitochondrial markers, which do not form aggregates. Hence, the true kinetics of fast ΔΨm changes might be underestimated [54, 86]. However, as we did not aim to resolve temporally any ΔΨm changes, but rather compared steady-state conditions or drug treatment endpoints, this should be of no concern. A true drawback of JC-1 is, however, that comparative ΔΨm analyses in more intact preparations are hardly possible, as the excessive background fluorescence of interstitial JC-1 monomers prevents a meaningful JC-1 staining in, for example, acute or organotypic tissue slices.

Nevertheless, JC-1 and its derivatives are the only ratiometric mitochondrial ΔΨm indicators, and as such, their fluorescence response is not affected by the extent of dye uptake and/or differences in cellular mitochondrial content. All of these apply, however, to other nonratiometric ΔΨm indicators (e.g., rhodamine 123 or TMRM), and it may markedly complicate data analyses, especially when these compounds are used in low-resolution approaches, such as flow-cytometry and cuvette-based spectrophotometric assays. Only recently, an improved variant of JC-1, termed JC-10, became available. It functions just as JC-1 but offers higher water solubility and an improved dynamic response range (see http://www.enzolifesciences.com/fileadmin/reports/Datasheet-ENZ-52305.pdf). Thus, it may prove more advantageous also for future emission-ratiometric 2-photon imaging applications of ΔΨm alterations in individual mitochondria or ΔΨm differences among various cell types and/or genotypes.

## 5. Conclusions

Focusing on astrocytes, we performed functional optical analyses on the subcellular level, to extend earlier findings on mitochondrial alterations and redox imbalance in RTT. As the entire study is based on dissociated cell cultures, it can only reflect the neonatal developmental stage. Nevertheless, an increased mitochondrial mass and more oxidized cytosolic redox conditions were already detectable in *Mecp2^−/y^* hippocampal astrocytes of presymptomatic mice. This genotypic difference in mitochondrial mass was obvious for absolute mitochondrial content, its normalization to cell size, and MitoTracker labeling. This confirms that also astrocytes undergo clear alterations already during the neonatal and presymptomatic stages of RTT, which further supports the hypothesis that mitochondrial alterations and the associated oxidative burden drive the progression of this neurodevelopmental disorder. Trolox did not mediate any adverse effects on mitochondria, which is certainly of interest for free-radical scavenger- and antioxidant-based pharmacotreatment concepts in RTT. More importantly, this free-radical scavenger successfully abolished the genotypic differences in mitochondrial content among WT and *Mecp2^−/y^* astrocytes and improved cytosolic redox balance especially in *Mecp2^−/y^* astrocytes. This identifies cellular redox imbalance as one of the mechanisms underlying the increased mitochondrial mass in *Mecp2^−/y^* astrocytes.
